# Construction of endophenotypes for complex diseases in the presence of heterogeneity

**DOI:** 10.1186/1471-2156-6-S1-S139

**Published:** 2005-12-30

**Authors:** Chien-Hsiun Chen, Chih-Ling Kuo, Michael CP Lin, Yu-Jen Liang, Cathy SJ Fann

**Affiliations:** 1 Institute of Biomedical Sciences, Academia Sinica, Taipei, Taiwan; 2 College of China Medicine, China Medicine University, Taichung, Taiwan; 3 Institute of Public Health, National Yang-Ming University, Taipei, Taiwan

## Abstract

Endophenotypes such as behavior disorders have been increasingly adopted in genetic studies for complex traits. For efficient gene mapping, it is essential that an endophenotype is associated with the disease of interest and is inheritable or co-segregating within families. In this study, we proposed a strategy to construct endophenotypes to analyze the Genetic Analysis Workshop 14 simulated dataset. Initially, generalized estimating equation models were employed to identify phenotypes that were correlated to the disease (affected status) in combination with the family structures in data. Endophenotypes were then constructed with consideration of heterogeneity as functions of the identified phenotypes. Genome scans on the constructed endophenotypes were carried out using family-based association analysis. For comparison, genome scans were also performed with the original affected status. The family-based association analysis using the endophenotypes correctly identified the same susceptible gene in about 80 of the 100 replicates.

## Background

Field diagnostic classification schemas are commonly used to evaluate subjects' development of complex disorders. For heterogeneous traits, subjects categorized as being affected may be trigged by different genetic or environmental components. Consequently, genetic analysis becomes more difficult when heterogeneity is embedded in such a poorly defined phenotype. Concepts using well defined endophenotypes had been applied to facilitate the process of gene mapping [1]. In general, an endophenotype may represent simpler clues to genetic effects than the disease status and hence can be applied to identify more homogeneous subgroups. On the other hand, a well defined endophenotype on the pathway may act as a biomarker for a more accurate assessment of complex disease or for an earlier diagnosis of late onset diseases. In this study, our goal was to construct endophenotypes that could more accurately identify genes susceptible to the complex trait of interest in the presence of heterogeneity.

## Methods

### Materials

Genetic Analysis Workshop 14 (GAW14) provided a simulated dataset with 100 replicates. Original phenotypic data of diseased families from four geographically diverse sites, Aipotu, Karangar, Danacaa, and New York City, were collected separately with varied criteria for diagnosis of Kofendrerd personality disorder (KPD). Subjects from these four groups differed in their living environment, life style, and ethnicity. In the simulation data, 100 nuclear families were generated for the first three groups and 50 extended large pedigrees were generated for the fourth group. Genomes with 10 chromosomes were constructed with a total of 917 single-nucleotide polymorphism (SNP) and 413 microsatellite markers. In this study, only SNP data was analyzed. In addition to a dichotomous KPD affected status, twelve binary phenotypes, labeling as *a*, *b*, *c*, ...,*l*, were given for each individual.

### Phenotype analysis using generalized estimating equation

Generalized estimation equations (GEE) were introduced by Liang and Zeger [2] as a method to estimate parameters of linear models when dealing with correlated data. If the correlation is not taken into account, the standard errors of the parameter estimates would not be valid and hypothesis testing results would not be applicable.

In the first part of this study, GEE with logit link functions was applied to test the difference among the four groups with respect to KPD (KPD~group). Each family was treated as a single unit; members of the same family were treated as replicates with equal correlations. A significant group effect may indicate that a possible heterogeneity of KPD or population structure exists in the pooled data. Correlations between KPD and 12 binary phenotypes were separately assessed using similar GEE models (KPD ~ *X, X *= *a*, *b*, *c*, ..., *l*). It may indicate heterogeneity of KPD if the four groups had different subsets of the 12 phenotypes in associated with KPD. GEE analysis was performed using SAS/GENMOD software (SAS Institute, Cary, NC, USA). For this study, a cut-off at a significant level 0.05 was used to define correlated phenotypes.

### Endophenotype construction

The second step was to construct endophenotypes. We defined two types of endophenotypes. The first was to take each correlated phenotype as an endophenotypes and the second was to derive endophenotypes from those correlated binary phenotypes with the Boolean operators "or" and "and". For example, suppose three phenotypes, *a*, *b*, and *c*, are significant in the first step. An endophenotype can be defined, using the Boolean operator "or", by the following rules: a subject will be categorized as "affected" if one of the three phenotypes is positive, and "unaffected" if the three phenotypes are negative. An endophenotype will advance to the next step if it is associated with the disease of interest. For this study, GEE with logit link functions was applied to examine the relationship between endophenotypes and KPD.

### Genome scans using family-based association analysis

The third step was to conduct genome scans using family-based association tests (FBAT). In general, FBAT methods were set to compute *p*-values by comparing test statistics for association to their conditional distributions given the minimum sufficient statistic under the null hypothesis for the genetic model, sampling plan, and population admixture [3]. In the study, single-point genome scans were carried out using computer software also named FBAT [3,4].

### Replicates and keys

The last step was to repeat the above analysis for the 100 replicates and estimate the test power, in terms of the ratio of hits at true susceptible loci detected by FBAT analysis using endophenotypes. The analysis was done without knowing the true locations of the disease loci, the simulation algorithms, and the parameter settings prior to the calculation of test power.

## Results

### Correlations between KPD and group, KPD and 12 phenotypes

In the initial GEE analysis, the group effect was significantly related to KPD. Data from four groups should not be combined without proper adjustment, therefore GEE models for detecting correlation between KPD and phenotypes were applied to each group separately. In most of the 100 replicates, only two phenotypes, *a *and *k*, showed significance (*p *< 0.05) for all groups consistently while Aipotu and NYC groups shared a common set of phenotypes related to the disease: *a*, *b*, *c*, *d*, *g*, *k*, and *l*. Table 1 shows the summary statistics of GEE analyses of 100 replicates.

### Construction and examination of endophenotypes

At the second stage, phenotypes *a *and *k *were chosen, in most of the 100 replicates, to construct four endophenotypes, *a*, *k*, (*a and k*), and (*a or k*). The four endophenotypes were also constructed for replicates in which phenotypes *a *and *k *were not significantly related to KPD. GEE analyses were carried out with model *KPD *~ *X*, where *X *= *a*,*k*, (*a and k*), (*a or k*), for the 100 replicates. Endophenotypes *a*, *k *and (*a or k*) were highly significantly related to KPD (*p*-values < 0.05 in all four groups of 100 replicates) while (*a and k*) varied across groups (Table 1). In addition, GEE analysis was performed to test the interaction between *group *and the four endophenotypes with respect to KPD. The interactions between *group *and *k *and between *group *and (*a or k*) were significant in 15 and 31 replicates, respectively, while the interactions between *group *and *a *and between *group *and (*a and k*) were significant in at least 70 of the 100 replicates. This might indicate that the endophenotypes *k *and (*a or k*) could be more appropriate in analyses with pooled data from the four groups.

**Figure 1:**
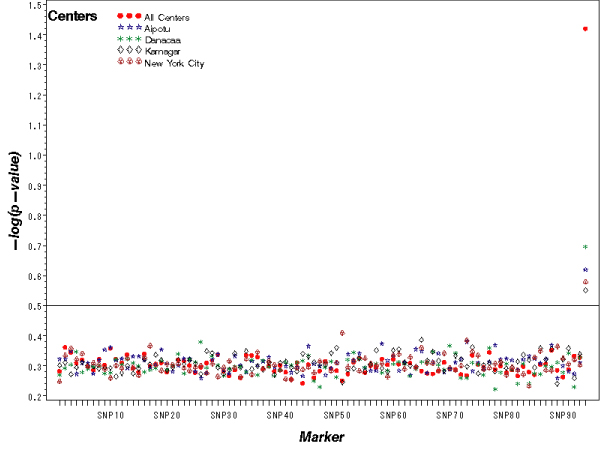
**Means of -log(*p*-value) of FBAT across chromosome 3. **The last SNP on chromosome 3 is the most significant locus.

Genome scans using FBAT in pooled data identified the same susceptible SNP at the end of chromosome 3 for endophenotypes *k *and (*a or k*) in 83 and 80 replicates, respectively. In addition, genome scans were performed with the two endophenotypes *k *and (*a or k*) for each group. Genome scan data shows higher means of -log (*p*-value) at the identified SNP in pooled data than in separated groups. Figure 1 shows that the most significant locus was found at the end of chromosome 3. No other SNPs were significantly related to *k *and (*a or k*) in more than 30 replicates. For comparison, genome scans were performed with the endophenotype (*a and k*) in the pooled data, but only 16 out of the 100 replicates identified the same SNP on chromosome 3.

## Discussion

In general, association analysis with pooled data from structured population might give spurious association [5]. On the other hand, the FBAT methods are quite robust even for samples from an admixed population [3]. Therefore, in this study the derived endophenotypes had higher means of -log (*p*-values) in pooled data while maintaining a moderate test power in genome scans using the FBAT method. We assumed that the 12 basic phenotypes were well established measurements and had no heterogeneity among groups. From the simulation algorithms stated in the "answer", the above assumption might not be true for all of the 12 phenotypes. Studies in using phenotypes with heterogeneity may further clarify this issue. From the revealed simulation algorithms, most of the 12 phenotypes had a high phenocopy rate of 30%, which could result in a lower power of using the endophenotypes based on them. An alternative to the construction of qualitative endophenotypes as in this study is to build quantitative endophenotypes as a scoring system for screening the target disease.

## Conclusion

In this study, we constructed endophenotypes when the trait of interest showed heterogeneity among sampling groups. The power to detect a true gene in FBAT analysis using the endophenotype remained moderate at 80%, despite the complexity of the underlying genetic models of the simulation data. Our method might be useful to derive endophenotypes for a cross-culture assessing instrument of KPD.

## Abbreviations

FBAT: Family-based association test

GAW14: Genetic Analysis Workshop 14

GEE: Generalized estimation equations

KPD: Kofendrerd personality disorder

SNP: Single-nucleotide polymorphism

## Authors' contributions

C-HC participated in study design and coordination and drafted the manuscript. C-LK carried out the GEE analysis of phenotypes, prepared data for genome scans, and helped to draft the manuscript. MCPL help to set up the GEE analysis, participated in study design, and helped to draft the manuscript. Y-JL participated in study design and performed the genome scan. CSJF conceived the study, participated in its design, and helped to draft the manuscript. All authors have read and approved the final manuscript.

## Figures and Tables

**Table 1:** Percentage of significant correlations between KPD and phenotypes in 100 replicates.

Phenotype	Aipotu	Danacaa	Karangar	New York City
a	100%	100%	97%	100%
b	100%	0%	100%	100%
c	100%	10%	0%	100%
d	100%	15%	0%	100%
e	0%	0%	0%	0%
f	0%	0%	0%	0%
g	100%	9%	0%	100%
h	0%	0%	0%	0%
i	7%	7%	3%	3%
j	6%	4%	3%	5%
k	100%	88%	100%	100%
l	92%	9%	95%	97%
(a or k)	100%	100%	100%	100%
(a and k)	15%	55%	31%	51%
